# Use of a BMI-independent biomarker-based prostate cancer risk score to identify and triage individuals at risk of prostate disease

**DOI:** 10.1038/s41598-025-13036-w

**Published:** 2025-07-24

**Authors:** Joanne Watt, Allister Irvine, Mary Jo Kurth, Laura Mooney, Gary Smyth, Hardev Pandha, John Lamont, Peter Fitzgerald, Le Roy Dowey, Mark W. Ruddock

**Affiliations:** 1 Randox Laboratories Ltd., 55 Diamond Road, Crumlin, Antrim, BT29 4QY UK; 2 Randox Health GB, 143-149 Great Portland Street, Marylebone, London, W1W 6QN UK; 3 Research Development and Innovations Department, Royal Surrey County Hospital NHS Foundation Trust, The Royal Surrey County Hospital, Guildford, GU2 7XX UK; 4 School of Biomedical Sciences, Ulster University, Coleraine, Londonderry, BT52 1SA UK

**Keywords:** Prostate cancer risk score, Triage, Prostate specific antigen, Body mass index, Prostate cancer, Prostate cancer, Cancer screening

## Abstract

Prostate cancer (PCa) is the second most common cause of cancer related deaths in men in the UK. A national screening programme for PCa does not exist due to the unsuitability of the total prostate specific antigen (tPSA) test which is not specific for PCa and has a high false positive rate. Serum tPSA was measured in *n* = 25,356 male Randox Health clients. A biomarker-based (tPSA, EGF, MCP-1, IL-8) prostate cancer risk score (PCRS) was then applied to a retrospective cohort (*n* = 1,142/25,356) of individuals to assess PCa risk. A comparative analysis between tPSA and PCRS indicated that 90.5% of the cohort were assigned low risk of PCa. Of those with an elevated PCRS, 67.8% (78/115) had a normal tPSA value based on tPSA age-adjusted cut-offs. In addition, we observed a significant negative correlation between increasing body mass index (BMI) in men with high BMI (≥ 30) and tPSA levels. No correlation was observed between BMI and PCRS. The tPSA test is potentially unsuitable for use in males with BMI ≥ 30. Use of PCRS could provide more accurate PCa risk stratification for males with BMI ≥ 30. Future assessment of the clinical utility of PCRS is warranted.

**Supplementary Information:**

The online version contains supplementary material available at 10.1038/s41598-025-13036-w.

## Introduction

Worldwide cases of prostate cancer (PCa) are projected to double from 1.4 million in 2020 to 2.9 million by 2040 ^1^. In the UK, PCa is the most common cancer and the second most common cause of cancer-related deaths in males^2^. The UK has a higher age standardised PCa mortality rate (11.8) per 100,000 of the male population, than in Ireland (9.9), France (8.2), USA (8.1), Spain (7.6) and Italy (6.6)^3^. Late detection of PCa contributes to its associated high mortality^1^. For example, of 180,000 men who were diagnosed with PCa between 2015 and 2019 in England, 16.4% were diagnosed with stage IV disease^4^. In the UK, there is disparity between the stage of PCa diagnosis where men living in areas with higher socioeconomic deprivation are 29% more likely to be diagnosed with stage IV disease^4^. There is also geographical variation in the stage of PCa at diagnosis. For example, in London 12.5% of men presented with stage IV PCa whereas in Scotland and Northern Ireland 35% and 20% of males, respectively, were diagnosed with stage IV PCa^5^. Five-year survival rates for males diagnosed with PCa at stage I, II and III are > 95% however, survival rates decrease to ~ 50% for those diagnosed at stage IV^6^.

There are various risk factors associated with PCa including age, ethnicity, and lifestyle^7^. Various studies have indicated that men with a higher body mass index (BMI) are also at an increased PCa mortality risk^7^. An analysis of UK Biobank data found that for every five-point increase in BMI, the risk of fatal PCa increased by 10%^8^; the underlying mechanism resulting in elevated risk is not well understood but may include late detection and diagnosis^8^.

Despite the high proportion of males diagnosed with late-stage cancer, PCa screening is not available in the UK. Currently, a positive total prostate specific antigen (tPSA) blood test can be used as an indicator of prostate-related issues^9^. Although the tPSA test is sensitive for detecting prostate-related problems, it is not specific enough to distinguish between PCa and other conditions including urinary tract infections, benign prostate enlargement or prostatitis^10^. Additionally, tPSA levels increase with age and there is limited consensus on which tPSA cut-off is best to differentiate between benign disease and malignancy^11^. While various studies have demonstrated that tPSA testing can be a useful PCa screening tool^10^, the low specificity of the test leads to high false positive rates^12^. For example, in the European Randomised study of Screening for Prostate Cancer, tPSA screening resulted in a relative mortality reduction of 20%^13^. However, > 75% of males referred for prostate biopsy, based on elevated tPSA, were negative for PCa^13^. Thus, screening for PCa using tPSA would potentially increase the number of individuals referred to secondary care for unnecessary and invasive procedures such as transrectal or transperineal ultrasound-guided prostate biopsy.

Many patients have clinically insignificant indolent PCa tumours and there is potential for overtreatment. Patients with clinically insignificant PCa are typically asymptomatic, and there is no impact on quality of life. In many cases, treatment of these tumours is unnecessary and complications resulting from overtreatment can be detrimental to patient health. A systematic review reported that screening for PCa using the tPSA test would increase the number of males per 1,000 who are diagnosed with PCa from 68 to 88^14^. However, it was expected that PCa in these individuals would not have caused harm, and lives would not be saved through screening^14^. It is important that any novel diagnostic tool can distinguish between clinically significant and clinically insignificant PCa.

Magnetic resonance imaging (MRI) can also be used to aid diagnosis of PCa and can reduce the number of patients referred for unnecessary biopsies by 27%^15^. MRI is typically performed on patients who have been referred based on an elevated tPSA level and/or prostate abnormalities (digital rectal exam). A recent clinical trial (VERDICT MRI) investigated the use of MRI as a screening tool for PCa independent of a tPSA test^16^. The study determined that of the 16% of individuals that screened positive who had a follow-up biopsy, only half of these individuals had clinically significant PCa. Significantly, over half of the individuals with clinically significant PCa had a tPSA level below 3ng/ml and thus would have been missed using tPSA screening^16^. However, MRI resources are limited and clinically significant PCa can remain undetected using MRI^17^. Additional tests are needed to indicate PCa risk and inform clinical decision making.

There are various tests which aim to improve PCa detection in individuals at risk including the Stockholm3 test, SelectMDx test, and 4Kscore^®^ test^18^. Use of these tests can improve risk stratification for patients who present with elevated tPSA levels and potentially reduce the need for unnecessary biopsies. However, these tests still require an initial tPSA test to identify at-risk patients and are more suited for use in secondary care risk stratification due to their costs. For example, in a review of the Stockholm3 test by the National Institute for Health and Care Excellence (NICE), the high cost per unit (£350) compared to tPSA testing in primary care (£27.75) was indicated as a potential limitation to adoption of the test in the National Health Service (NHS)^19^.

Previously we tested 19 serum biomarkers in patients who were referred into secondary care for PCa investigations. Through logistic LASSO regression analyses we identified a combination of four biomarkers (tPSA, epidermal growth factor (EGF), monocyte chemoattractant protein-1 (MCP-1) and interleukin-8 (IL-8)), which could be used to help triage symptomatic individuals at risk of PCa into low- and high-risk categories and improve clinical decision making^20^. The combination of these four biomarkers was used to calculate a novel biomarker-based algorithm (prostate cancer risk score (PCRS)).

The aim of the current observational study was to apply the PCRS to data obtained from a cohort of males, who attended Randox Health Clinics for health checks, and to investigate the concordance between PCRS and tPSA. Furthermore, the impact of BMI on tPSA, PCRS, and male hormonal biomarkers, was also investigated.

## Methods

### Sample population

The cohort in this study involved *n* = 25,356 samples from *n* = 20,793 UK males who had attended a Randox Health Clinic for a health check or submitted postal samples between January 2019 and December 2023. Venous blood samples were collected and processed at the clinic whereas capillary samples collected at home were posted and processed at Randox Clinical Laboratory Services (RCLS) (Antrim, Northern Ireland, UK; ISO17025 accredited) for biomarker level determination. Informed consent from all study participants was recorded digitally through the Randox portal, and all individual data were anonymised. The study was reviewed and approved by Ulster University School of Biomedical Sciences Ethics Filter Committee (Project Number: FCBMS-24-187). All methods were carried out in accordance with relevant guidelines and regulations and were conducted in accordance with the Declaration of Helsinki. It was not possible to involve patients or the public in the design, or conduct, or reporting, or dissemination plans of our research. For patients who attended a Randox Health clinic, patient measurements (height (cm), weight (kg), waist and hip circumference (cm)) were recorded by clinic staff.

### Sample analyses and application of prostate cancer risk score (PCRS)

Serum biomarker levels were determined by RCLS. Albumin levels were analysed on a RX Daytona+. Follicle stimulating hormone (FSH), luteinising hormone, oestradiol, prolactin, sex hormone binding globulin (SHBG), testosterone, and tPSA were analysed on a Roche cobas analyser. EGF, MCP-1 and IL-8 were analysed using Biochip Array Technology (High Sensitivity Cytokine Array I) on an Evidence Investigator analyser (Randox Laboratories Ltd., Crumlin, UK)^21^ (see Supplementary Materials for more information). Analyte limits of detection (LOD) were: albumin (3.2 g/l), EGF (1.04pg/ml), FSH (0.3IU/ml), IL-8 (0.36pg/ml), luteinising hormone (0.3IU/ml), MCP-1 (3.53pg/ml), oestradiol (5pg/ml), prolactin (0.094ng/ml), SHBG (0.8nmol/l), testosterone (2.50ng/dl), and tPSA (0.010ng/ml). Where analyte readings were below the LOD, they were recorded at 90% of the LOD^22^. Free testosterone was calculated using testosterone, SHBG and albumin levels using the following equation^23^.

$$\:Free\:testosterone\:(mol/L)=[-b+\:\surd\:({b}^{2}+4a\:\times\:\:testosterone\left)\right]\:/\:2a$$

where $\:a$ = $\:{k}_{at}+\:{k}_{t}+\left({k}_{at}\:\times\:\:{k}_{t}\right)\:\times\:\left(SHBG+albumin-testosterone\right)$; $\:b$ = $\:1+\:{k}_{t}\:\times\:\:SHBG+\:{k}_{at}\:\times\:albumin-\left({k}_{at}+\:{k}_{t}\right)\times\:testosterone$; $\:{k}_{at}$ = 3.6 × 10^4^ L/mol; $\:{k}_{t}$ = 10 × 10^8^ L/mol.

An age-adjusted tPSA cut-off was used to determine individuals at risk of PCa based on their tPSA alone, according to NICE^9^: <50 years > 2.5ng/ml tPSA, 50–59 years > 3.5ng/ml tPSA, 60–69 years > 4.5ng/ml tPSA, 70–79 years > 6.5ng/ml, and > 79 years > 6.5ng/ml tPSA. PCRS biomarker readings were available for *n* = 1,142 samples (*n* = 992 males). Individual samples were screened for PCa risk using the following PCRS logistic regression:

$$\:PCRS=c+m1\left(EGF\right)+m2\left({log}_{10}IL\text{-}8\right)+m3\left({log}_{10}MCP\text{-}1\right)+m4\left({log}_{10}tPSA\right)$$

where $\:c$ = −8.92652, $\:m1$ = 0.01002, $\:m2$ = −1.52428, $\:m3$ = 3.95772, $\:m4$ = 1.31471 and with a PCRS cut-off of 0.05385012.

### Body mass index (BMI)

Where an individual’s height (cm) and weight (kg) information was available, BMI (kg/m^2^) was calculated^24^. Where waist circumference (WC) and hip circumference measurements were available, waist-hip ratio was calculated.

### Statistical analyses

All analyses and graphs were completed using R, version 4.3.1^25^. Calculation of the PCRS has been described previously^20^. All biomarker levels except EGF were not normally distributed and were log_10_ transformed. Pearson correlation coefficients were calculated for all biomarkers and BMI. Stars of significance indicate: ** p-value < 0.01, **** p-value < 0.0001.

## Results

### tPSA levels in the total sample and biomarker cohorts

**Fig. 1:**
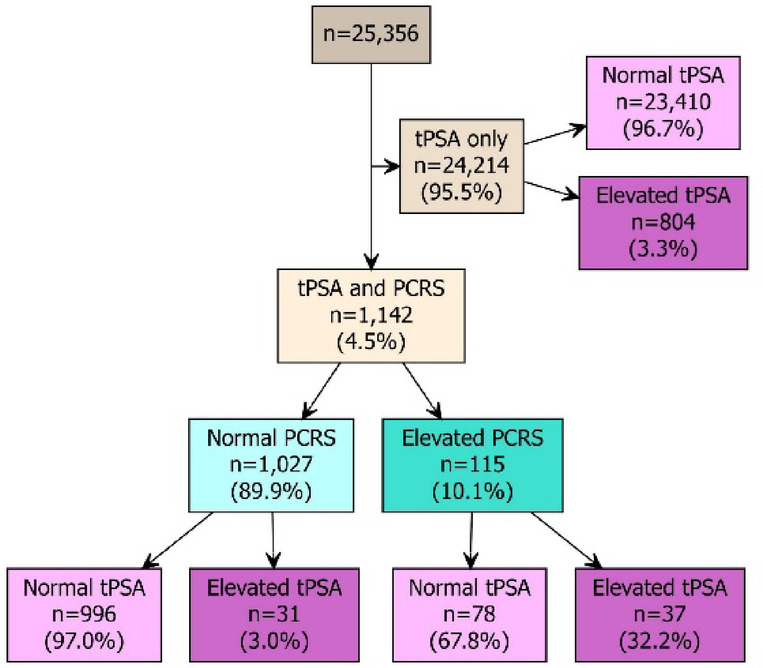
Breakdown of total sample cohort based on total prostate specific antigen (tPSA) level and prostate cancer risk score (PCRS) (tPSA, epidermal growth factor (EGF), monocyte chemoattractant protein-1 (MCP-1) and interleukin-8 (IL-8)).

**Fig. 2:**
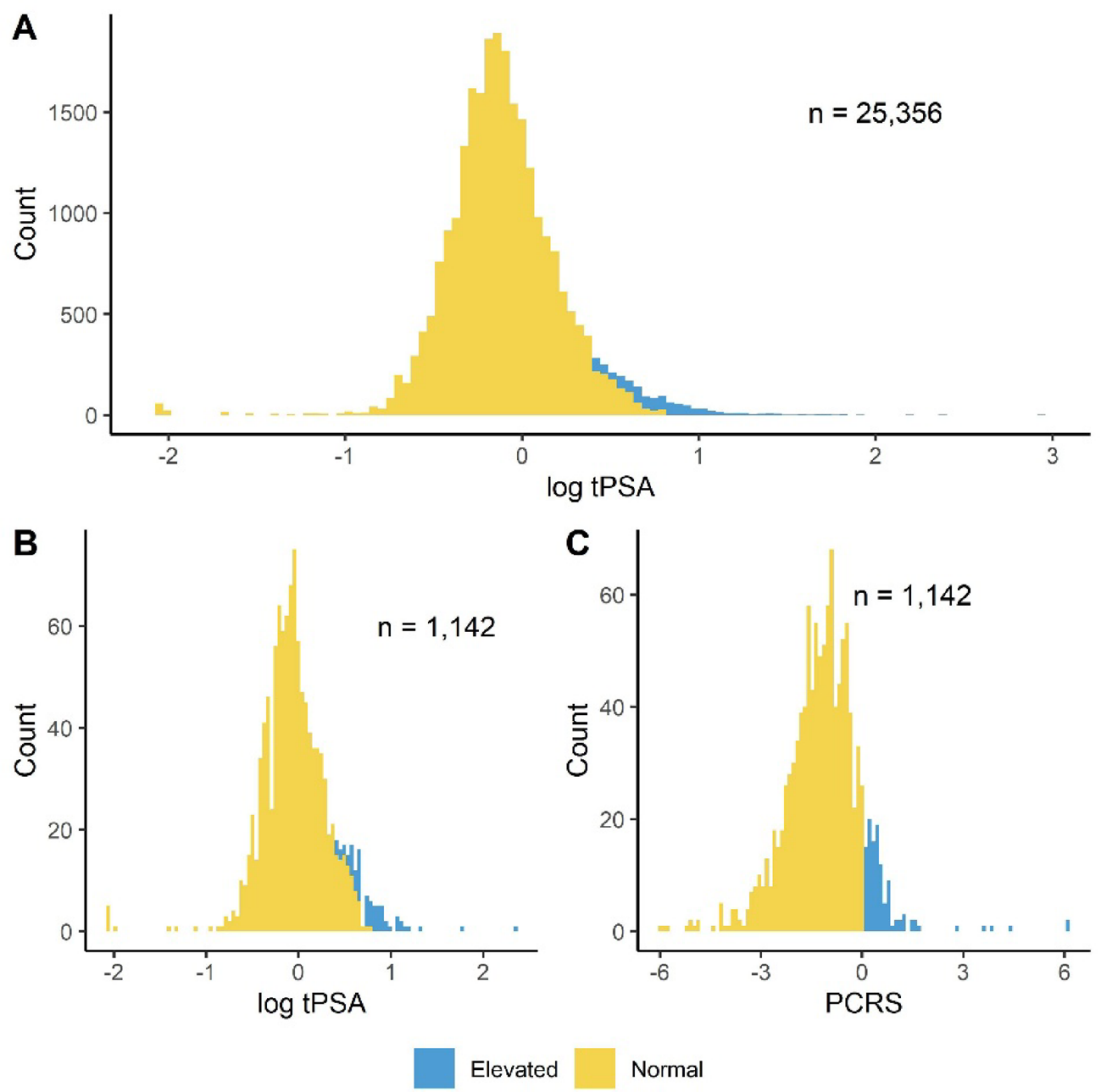
The distribution of total prostate specific antigen (tPSA) and prostate cancer risk score (PCRS) values across the total sample and biomarker cohorts. **(A)** Distribution of log_10_ tPSA values in the total sample cohort (*n* = 25,356). **(B)** Distribution of log_10_ tPSA values in the biomarker cohort (*n* = 1,142). **(C)** Distribution of PCRS values in the biomarker cohort (*n* = 1,142). Samples which exceeded the age-adjusted tPSA (based on NICE guidelines) and PCRS cut-offs (0.05385012), are shown in blue.

The patient demographics for the total sample cohort (*n* = 25,356) included in this study are described in Table 1. To assess PCa risk, 95.5% (24,214/25,356) of the total sample cohort had tPSA measured only. Of these, 96.7% (23,410/24,214) had a normal age-adjusted tPSA (Figs. 1 and 2A), with 3.3% (804/24,214) deemed at risk of prostate-related issues according to NICE guidelines^9^. However, 4.5% (1,142/25,356) of the total cohort also had additional biomarkers (EGF, MCP-1, IL-8) measured with tPSA, to assess PCa risk as part of their Randox Health check (biomarker cohort, *n* = 1,142). In the biomarker cohort, 6.0% (68/1,142) would have been assigned at risk based on an age-adjusted tPSA cut-off (Fig. 2B).

**Table 1:** Total sample demographics.

*Variables*	*n*	*Mean ± SD*	*Median (range)*
**Age (years)**	25,356	45.5 ± 13.5	43.9 (18.0-95.7)
**Height (cm)**	17,283	178.2 ± 6.8	178.0 (143.0-210.0)
**Weight (kg)**	17,269	85.7 ± 15.1	83.8 (43.7- 199.4)
**Waist circumference (cm)**	17,238	93.2 ± 12.0	92.0 (52.5–199.0)
**Hip circumference (cm)**	17,215	102.8 ± 8.1	102.0 (60.0-193.0)
**Waist-hip ratio**	17,214	0.9 ± 0.07	0.9 (0.6–1.9)
**Body mass index (BMI)**	17,262	27.0 ± 4.4	26.3 (14.6–59.5)
**tPSA (ng/ml)**	25,356	1.2 ± 6.4	0.8 (0.04–883.0)

### PCRS in the biomarker cohort

When the PCRS was applied, 89.9% (1,027/1,142) of the biomarker cohort had a normal PCRS and 10.1% (115/1,142) had an elevated PCRS (Figs. 1 and 2C). In samples from the biomarker cohort with a normal PCRS, 97.0% (996/1,027) were also designated normal based on tPSA. The remaining 3.0% (31/1,027) of samples had an elevated tPSA. In samples from the biomarker cohort with an elevated PCRS, 67.8% (78/115) had a normal tPSA and 32.2% (37/115) had an elevated tPSA (Fig. 1).

Out of the total biomarker cohort (*n* = 1,142), the tPSA and PCRS results were non-concordant in 9.5% (109/1,142) of samples; 2.7% (31/1,142) of samples had a normal PCRS with an elevated tPSA and 6.8% (78/1,142) of samples had an elevated PCRS and a normal tPSA level.

### Effect of body mass index (BMI) on PCa biomarkers from the PCRS

BMI information was available for *n* = 17,262 individual samples (Table 1) which were classified into four categories according to NICE guidelines^24^: underweight (BMI < 18.5), healthy weight (BMI 18.5–24.9), overweight (BMI 25-29.9) and obesity (BMI ≥ 30). The percentage in each category were: 0.5% (92/17,262) underweight, 35.0% (6,049/17,262) normal weight, 44.4% (7,672/17,262) with overweight, and 20.0% (3,449/17,262) with obesity. Note that BMI is unable to distinguish between muscle and fat content and some of those classified as overweight and obese may have low body fat and higher muscle mass^26^. For the purposes of grouping samples in this study, all individuals with a BMI ≥ 30 were classified with obesity regardless of muscle/fat distribution.

In the biomarker cohort (*n* = 1,142), BMI information was available for 52.4% (598/1,142) of individual samples; 0.2% were classified with underweight (1/598), 29.1% (174/598) healthy weight, 49.0% (293/598) with overweight and 21.7% (130/598) with obesity. Across all BMI categories, there was a high level of concordance (> 90%) between the tPSA and PCRS results (Table 2). There were 1.2% (7/598) of samples who had a normal PCRS but an elevated tPSA level across the BMI categories (Table 2). Notably, as BMI increased, there was an increasing proportion of samples with normal tPSA and an elevated PCRS. Indeed, 5.7% (10/175), 7.8% (23/293) and 8.5% (11/130) of samples in the healthy weight, overweight and obesity categories, respectively, were identified at risk of PCa based on their elevated PCRS while having a normal tPSA level.

**Table 2:** Concordance of tPSA and PCRS across different BMI categories of men.

A) Healthy weight (BMI 18.5–24.9; *n* = 174)		
	Normal PCRS	Elevated PCRS
Normal tPSA	157 (90.2%)	10 (5.7%)
Elevated tPSA	2 (1.1%)	5 (2.9%)

B) Overweight (BMI 25-29.9; *n* = 293)		
	Normal PCRS	Elevated PCRS
Normal tPSA	261 (89.1%)	23 (7.8%)
Elevated tPSA	4 (1.4%)	5 (1.7%)

C) Obesity (BMI ≥ 30; *n* = 130)		
	Normal PCRS	Elevated PCRS
Normal tPSA	115 (88.5%)	11 (8.5%)
Elevated tPSA	1 (0.8%)	3 (2.3%)

**Fig. 3:**
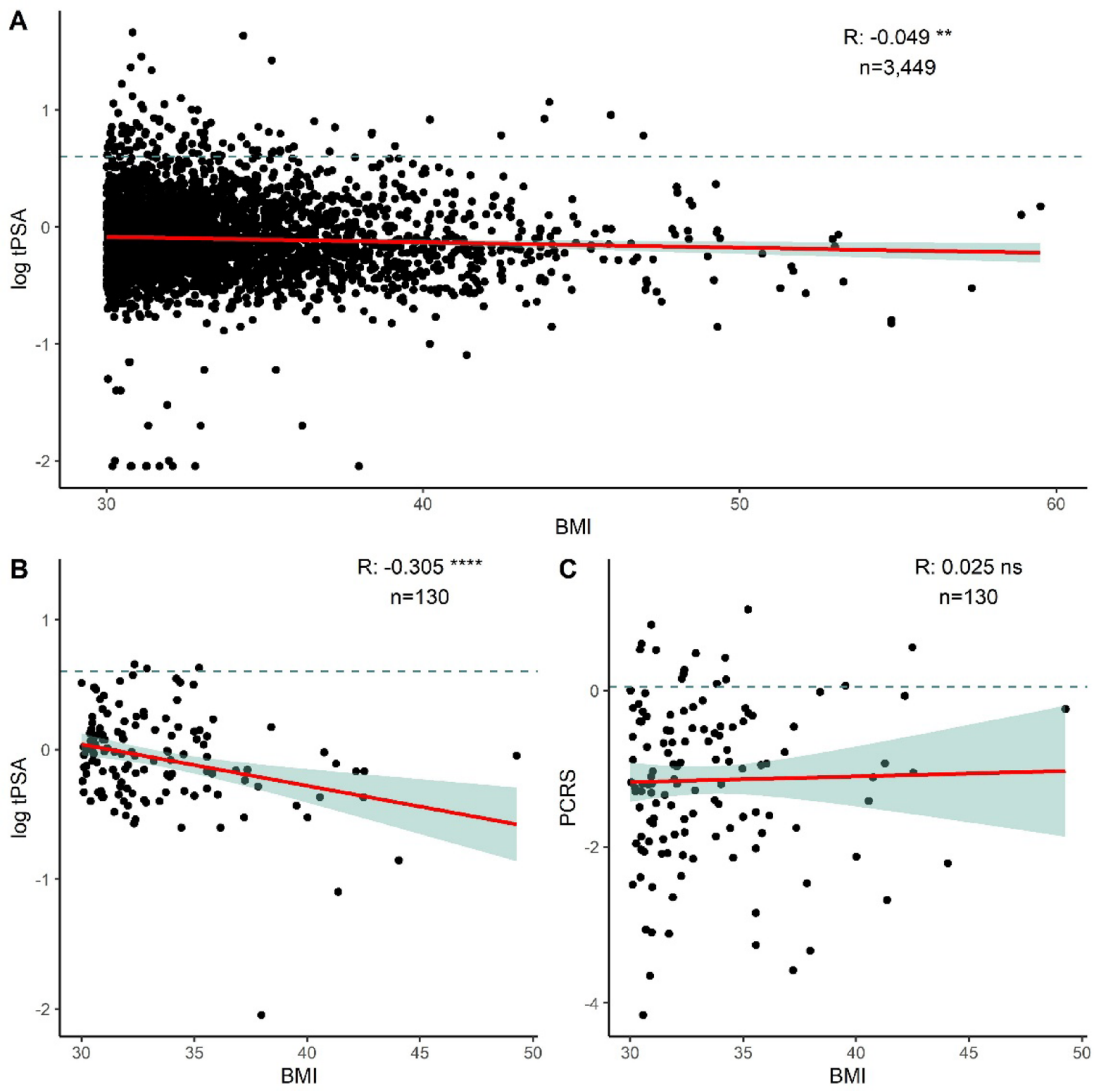
Correlations between body mass index (BMI, kg/m^2^), log_10_ total prostate specific antigen (tPSA) and prostate cancer risk score (PCRS) in individuals classified as obese (BMI ≥ 30). (**A**) tPSA versus BMI in the obese sample cohort (n = 3,449) (**B**) tPSA versus BMI in the obese biomarker cohort (n = 130) (**C**) PCRS versus BMI in the obese biomarker cohort (n = 130). Red line indicate correlation trendline. Green shaded area indicate 95% confidence interval. Dashed line in (**A**) and (**B**) represent tPSA level of 4ng/ml (log_10_ = 0.60206) and in (**C**) represent PCRS cut-off of 0.05385012. ** indicates p-value < 0.01, **** p-value < 0.0001.

In all individual samples with BMI information which were classified with obesity (BMI ≥ 30; *n* = 3,449/25,356), we observed a small but significant negative correlation (*n* = 3,449, *r* = −0.049, p-value = 0.0038; Fig. 3A) between tPSA and BMI. This association was also consistent across the smaller cohort of BMI ≥ 30 individuals with biomarker measurements (*n* = 130, *r* = −0.305, p-value = 0.0001; Fig. 3B). In the BMI ≥ 30 biomarker cohort (*n* = 130), we also observed a significant positive correlation between levels of EGF and increasing BMI (*n* = 130, *r* = 0.212, p-value = 0.016). There were no significant correlations between MCP-1 and BMI (*n* = 130, *r* = 0.09, p-value = 0.31) or IL-8 and BMI (*n* = 130, *r* = −0.035, p-value = 0.69) in the BMI ≥ 30 biomarker cohort (*n* = 130). Notably, there was no significant correlation between PCRS and BMI (*n* = 130, *r* = 0.025, p-value = 0.78; Fig. 3C) in the BMI ≥ 30 biomarker cohort (*n* = 130).

### Effect of BMI on hormonal biomarkers

**Fig. 4:**
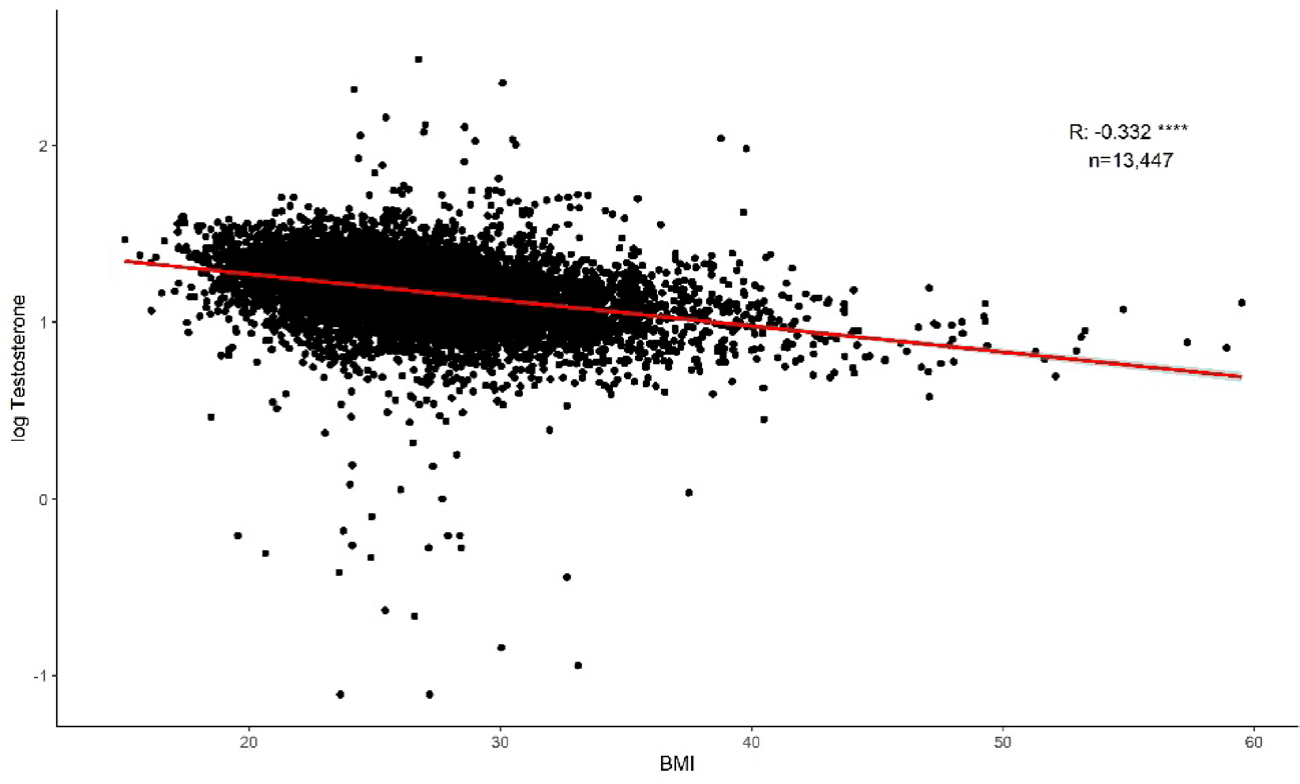
Correlations between body mass index (BMI) and log_10_ testosterone. Red line indicate correlation trendline. Green shaded area indicate 95% confidence interval. **** p-value < 0.0001. Other male hormone biomarker correlations (SHBG, free testosterone, oestradiol, prolactin, FSH and luteinising hormone) are shown in Supplementary Fig. 1.

In the total sample cohort (*n* = 25,356), the association between BMI and male hormonal biomarkers was investigated where data were available. We observed a significant negative correlation between increasing BMI and testosterone (*n* = 13,447, *r* = −0.332, p-value < 0.0001; Fig. 4), SHBG (*n* = 13,047, *r* = −0.286, p-value < 0.0001; Supplementary Fig. 1 A), and free testosterone (*n* = 5,414, *r* = −1.142, p-value < 0.0001; Supplementary Fig. 1B). There was a significant positive correlation between increasing BMI and oestradiol levels (*n* = 7,022, *r* = 0.077, p-value < 0.0001; Supplementary Fig. 1 C). There were no significant correlations between increasing BMI and prolactin (*n* = 6,808, *r* = 0.006, p-value = 0.728; Supplementary Fig. 1D), FSH (*n* = 6,176, *r* = 0.010, p-value = 0.565; Supplementary Fig. 1E) or luteinising hormone (*n* = 6,136, *r* = −0.019, p-value = 0.263; Supplementary Fig. 1 F).

## Discussion

The aim of the current study was to apply the PCRS to data obtained from a cohort of males who had visited Randox Health Clinics for health checks or posted samples from a home collection kit for prostate health testing. Another aim of the study was to investigate the effect of BMI on tPSA, inflammatory biomarkers in the PCRS and male hormone biomarkers.

In this study, we utilised a PCRS algorithm which incorporates tPSA and multiple inflammatory biomarkers (EGF, MCP-1, and IL-8). This combination of biomarkers has been shown previously to have an improved ability to predict PCa risk when compared to tPSA^20^. When the PCRS was applied to the cohort in this study, there was high agreement between tPSA and the PCRS (90.5%) in assigning the majority of samples as low risk for PCa. The cohort in this study included a high proportion of younger men (mean age 45.5 years) which are typically at lower risk of PCa^7^. However, 9.5% of the PCRS results were not concordant with the tPSA results; it is known that tPSA is not sensitive or specific enough to distinguish PCa from other prostate-related issues^10^.

We observed a negative correlation between tPSA and increasing BMI, which agrees with previous results^27^. Moreover, a recent systematic review determined that tPSA levels are on average, lower by 3.4% and 12.9% in men with overweight and obesity, respectively^28^. Notably, the prevalence of obesity is increasing globally^29^and in men with obesity, this negative association between tPSA and BMI could result in a higher proportion of false negative tPSA results leading to a delay in a potential PCa diagnosis. Moreover, in a review of the Prostate, Lung, Colorectal and Ovarian Cancer Screening Trial, males with higher BMIs had a lower risk of PCa diagnosis but a higher risk of PCa mortality^30^. Previous studies have suggested that tPSA cut-offs could be adjusted based on BMI^31^ however, studies have indicated that using a BMI-adjusted tPSA value does not improve the performance of the tPSA test^32^. Significantly, in the current study, we demonstrate that the PCRS biomarker-based algorithm is independent of changes in BMI.

It is important to note although BMI is a commonly used indicator of obesity in clinical practice, its usage is controversial^33^; alternative measures of adiposity have been suggested. WC measurements have been recommended as a measure of abdominal obesity^34^. A WC measurement of ≥ 102 cm in Caucasian males is considered very high risk of heart and circulatory diseases^35^. We observed the same significant correlations in increasing WC and tPSA in individuals with a WC ≥ 102 cm (Supplementary Fig. 2). Similar, to BMI, the PCRS was also independent of WC and thus could be a more accurate test for determining PCa risk in males with high BMI (≥ 30) and/or WC (≥ 102 cm).

Two mechanisms have been suggested for reduced serum tPSA levels observed in obese males: hemodilution (due to high BMI individuals having larger plasma volume) or low serum testosterone^36^. We observed a negative correlation between increasing BMI and serum testosterone, free testosterone and SHBG. In contrast, we observed a positive correlation between increasing BMI and serum oestradiol. These observations also agree with previous studies^37^. Testosterone stimulates the production of tPSA and testosterone replacement therapy in severely hypogonadal men increases serum tPSA levels^38^. In patients diagnosed with PCa, low tPSA has been shown to be predictive of low testosterone levels^39^. Measurement of male hormones such as testosterone in individuals at risk of PCa could inform clinical decision making when diagnosing and treating PCa.

For PCa screening to be effective it is essential to identify accurate methods to detect individuals at risk. Ongoing clinical trials such as the UK TRANSFORM PCa screening trial^40^ will be critical in assessing the efficacy of PCa screening. Similarly, further evaluation of the PCRS, as a tool to risk-stratify males at risk of PCa, will determine the effectiveness of the biomarker-based algorithm for detecting clinically significant PCa in clinical practice. Future studies should also evaluate the feasibility and cost-effectiveness of implementing measurement of additional biomarkers (IL-8, EGF and MCP-1) and application of the PCRS algorithm into clinical practice. Recently, Randox Laboratories have developed a multiplex test (Prostate Cancer Risk Biochip) which can simultaneously measure the levels of IL-8, EGF, MCP-1 and tPSA from a single serum sample. Evaluation and the feasibility of using the Prostate Cancer Risk Biochip and the PCRS within a clinical setting (primary and/or secondary care) is warranted.

## Conclusion

When PCa is detected early (stages I-III), five-year survival rates are high. Early detection is therefore critical to reduce the high mortality rate associated with late stage PCa. Large scale PCa screening programs require improved diagnostics to identify patients at risk of clinically significant cancer. Currently, the tPSA test, used as an indicator of prostate-related problems, lacks specificity and sensitivity for PCa. This study has highlighted that tPSA levels are negatively correlated with increasing BMI and thus tPSA testing may be inaccurately assigning high BMI individuals as low PCa risk. Significantly, high BMI individuals are also at greater risk of fatal PCa. We also demonstrate use of a BMI-independent PCRS which could be more appropriate to ensure high BMI individuals are not missed by tPSA testing. The PCRS could potentially provide increased information to clinicians which will allow them to make more informed clinical decisions. However, this requires validation in a longitudinal cohort with known clinical outcomes.

### Limitations of the study

There were limitations associated with this retrospective observational study. Data was not available reporting the clinical outcomes or the medication status of the males included in the cohort. There were also a limited number of samples in the high BMI (≥ 30) biomarker cohort (*n* = 130) and so further studies should include a higher number of high BMI (> 30) samples to confirm the biomarker associations. Furthermore, the mean age of the study cohort was younger (45.5 years) than individuals typically diagnosed with PCa. To clinically validate the PCRS, a confirmatory study comparing the accuracy of the PCRS to tPSA in a cohort with known biopsy/clinical outcomes is warranted.

## Electronic supplementary material

Below is the link to the electronic supplementary material.

Supplementary Material 1

## Data Availability

The datasets used and/or analysed during the current study are available from the corresponding author on reasonable request.
