# Supplementary material for: Use of a BMI-independent biomarker-based prostate cancer risk score to identify and triage individuals at risk of prostate disease
Source: Sci Rep. 2025 Jul 24;15:26864. doi: 10.1038/s41598-025-13036-w (PMC12287467; doi:10.1038/s41598-025-13036-w)

**Supplementary Materials**

**
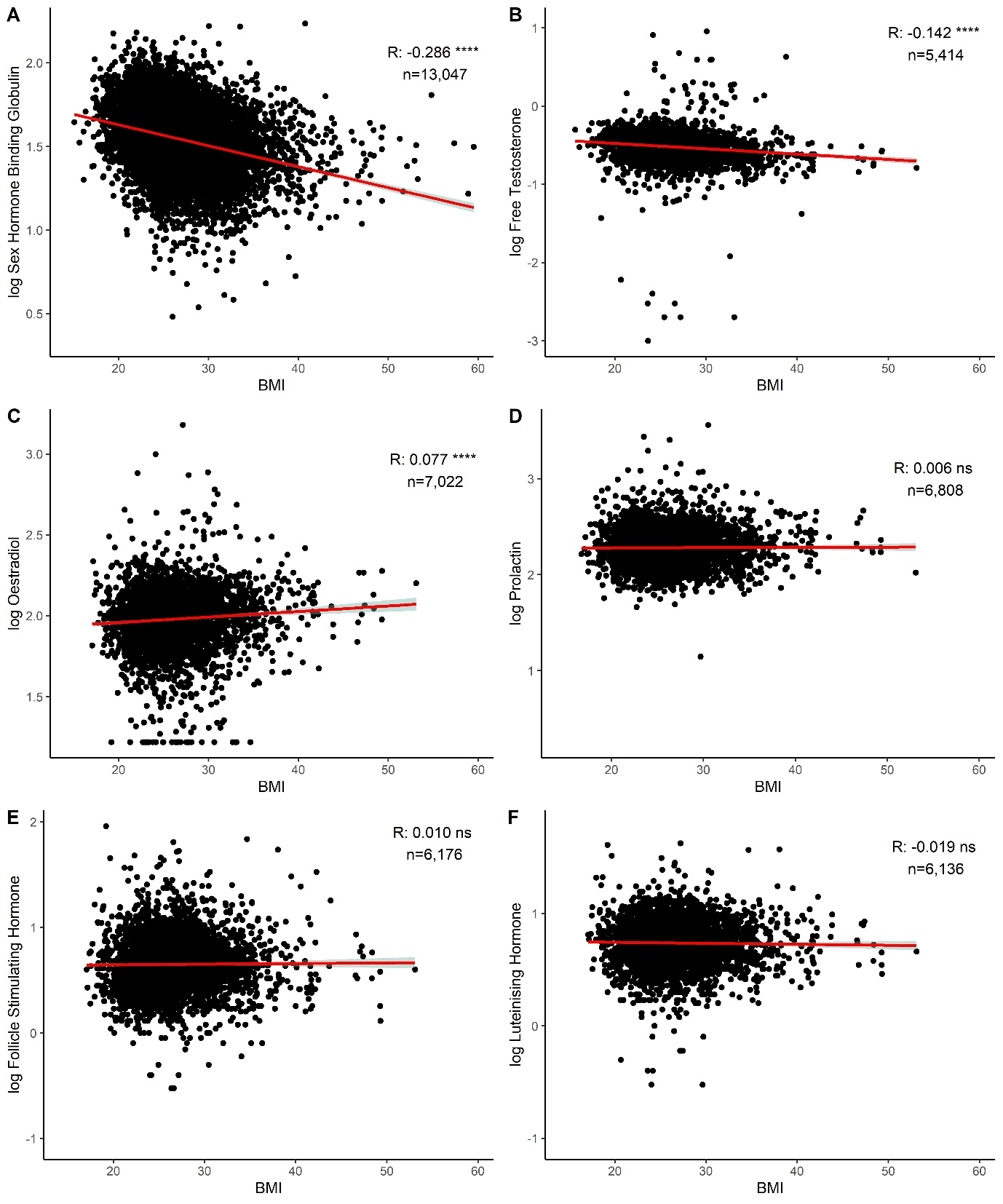
**

**Supplementary Figure 1: Correlations between body mass index (BMI), and male hormone biomarkers. A)** log_10_ sex hormone binding globulin versus BMI (n=13,047), **B)** log_10_ free testosterone versus BMI (n=5,414), **C)** log_10_ oestradiol versus BMI (n=7,022), **D)** log_10_ prolactin versus BMI (n=6,808), **E)** log_10_ follicle stimulating hormone (n=6,176), **F)** log_10_ luteinising hormone versus BMI (n=6,136). Red line indicate correlation trendline. Green shaded area indicate 95% confidence interval. **** p-value <0.0001.


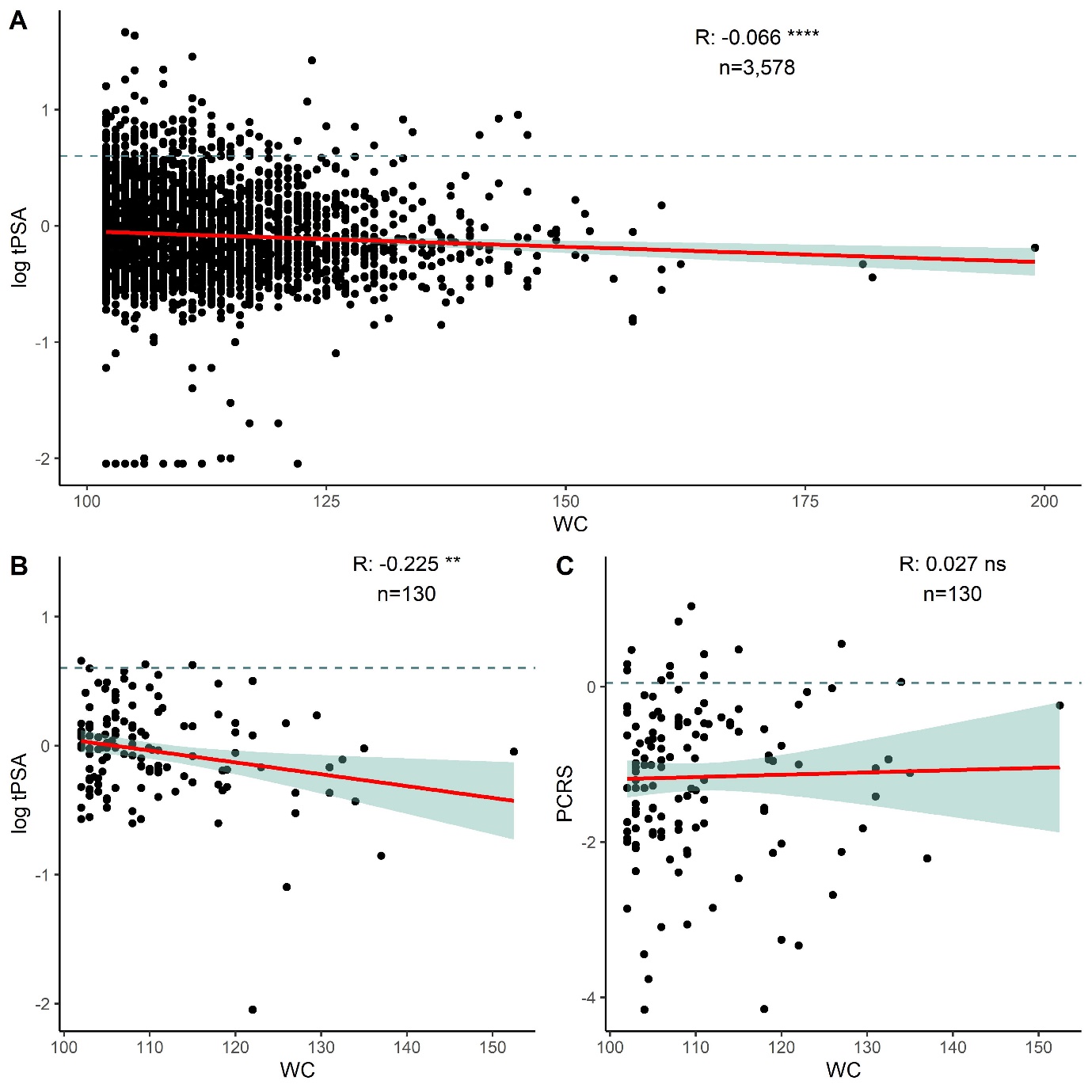


**Supplementary Figure 2: Correlations between waist circumference (WC), log_10_ total prostate specific antigen (tPSA) and prostate cancer risk score (PCRS) in very high-risk WC individuals (WC ≥102cm**). **A)** log_10_ tPSA versus WC in very high-risk WC individuals (n=3,578) **B)** log_10_ tPSA versus in very high-risk WC individuals with biomarker measurements (n=130) **C)** PCRS versus WC in very high-risk WC individuals with biomarker measurements (n=130). Red line indicate correlation trendline. Green shaded area indicate 95% confidence interval. Dashed line in **A)** and **B)** represent tPSA level of 4ng/ml (log_10_ = 0.60206) and in **C)** represent PCRS cut-off of 0.05385012. ** indicates p-value <0.01, **** p-value <0.0001.

**Supplementary Methods:**

The inflammatory biomarkers involved in the PCRS (monocyte chemoattractant protein-1 (MCP-1), interleukin-8 (IL-8) and epidermal growth factor (EGF)) were measured on High Sensitivity Cytokine Array I (EV 3623).

IL-8 is standardised against the NISBC 1st International Standard 89/520

EGF is standardised against NISBC/WHO 1st International Reference Reagent 91/550,

MCP-1 was standardised to gravimetrically measured human recombinant antigen.

The inter-assay variability for the inflammatory biomarkers were determined by assaying 20 replications of three levels of samples and are as follows:

IL-8:


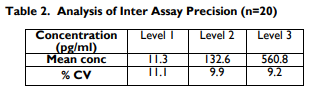


MCP-1:


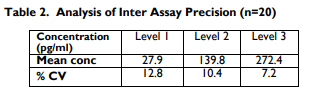


EGF:


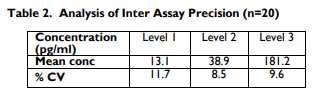

Supplement: Supplementary file 1 — Supplementary Material 1 [file 41598_2025_13036_MOESM1_ESM.docx]
